# Aerobic exercise improves episodic memory in late adulthood: a systematic review and meta-analysis

**DOI:** 10.1038/s43856-022-00079-7

**Published:** 2022-02-17

**Authors:** Sarah L. Aghjayan, Themistokles Bournias, Chaeryon Kang, Xueping Zhou, Chelsea M. Stillman, Shannon D. Donofry, Thomas W. Kamarck, Anna L. Marsland, Michelle W. Voss, Scott H. Fraundorf, Kirk I. Erickson

**Affiliations:** 1 Department of Psychology, University of Pittsburgh, Pittsburgh, PA USA; 2 Center for the Neural Basis of Cognition, University of Pittsburgh, Pittsburgh, PA USA; 3 Department of Psychology, Carnegie Mellon University, Pittsburgh, PA USA; 4 Department of Biostatistics, University of Pittsburgh, Pittsburgh, PA USA; 5 Department of Psychological and Brain Sciences, University of Iowa, Iowa City, IA USA; 6 Exercise Science, College of Science, Health, Engineering and Education, Murdoch University, Perth, WA Australia; 7 PROFITH “PROmoting FITness and Health Through Physical Activity” Research Group, Sport and Health University Research Institute (iMUDS), Department of Physical and Sports Education, Faculty of Sport Sciences, University of Granada, 18071 Granada, Spain

**Keywords:** Hippocampus, Randomized controlled trials

## Abstract

**Background:**

Aerobic exercise remains one of the most promising approaches for enhancing cognitive function in late adulthood, yet its potential positive effects on episodic memory remain poorly understood and a matter of intense debate. Prior meta-analyses have reported minimal improvements in episodic memory following aerobic exercise but have been limited by restrictive inclusion criteria and infrequent examination of exercise parameters.

**Methods:**

We conducted a meta-analysis of randomized controlled trials to determine if aerobic exercise influences episodic memory in late adulthood (*M* = 70.82 years) and examine possible moderators. Thirty-six studies met inclusion criteria, representing data from 2750 participants.

**Results:**

Here we show that aerobic exercise interventions are effective at improving episodic memory (Hedges’*g* = 0.28; *p* = 0.002). Subgroup analyses revealed a moderating effect of age (*p* = 0.027), with a significant effect for studies with a mean age between 55–68 but not 69–85. Mixed-effects analyses demonstrated a positive effect on episodic memory among studies with a high percentage of females (65–100%), participants with normal cognition, studies reporting intensity, studies with a no-contact or nonaerobic physical activity control group, and studies prescribing >3900 total minutes of activity (range 540–8190 min).

**Conclusions:**

Aerobic exercise positively influences episodic memory among adults ≥55 years without dementia, with larger effects observed among various sample and intervention characteristics—the clearest moderator being age. These results could have far-reaching clinical and public health relevance, highlighting aerobic exercise as an accessible, non-pharmaceutical intervention to improve episodic memory in late adulthood.

## Plain Language Summary

One of the earliest memory systems to decline with increasing age is episodic memory. Episodic memory is the remembrance of past personal events and experiences. Given a lack of pharmaceutical treatments to prevent or reverse this deterioration, it is important to investigate non-pharmaceutical methods for promoting the integrity of episodic memory. It remains unclear whether aerobic exercise induces changes in episodic memory. This study examined the effects of aerobic exercise randomized controlled trials on episodic memory in older adults without dementia and assessed whether the effects depend on the characteristics of the sample and intervention. We found that aerobic exercise positively influences episodic memory, with larger effects observed among various sample and intervention characteristics. These results highlight aerobic exercise as an accessible, non-pharmaceutical intervention to improve episodic memory in late adulthood.

## Abstract (web summary)

Aghjayan et al. conduct a systematic review and meta-analysis of randomised controlled trials on the effect of aerobic exercise on episodic memory in late adulthood. The results demonstrate that exercise interventions improve episodic memory, with age being one moderator of this effect.

## Introduction

One of the earliest cognitive domains to decline with increasing age and with Alzheimer’s disease (AD) is episodic memory (EM)^1,2^. Given that ~6.2 million adults ≥65 years in the United States have AD^3^ and the population of adults ≥65 years is projected to reach 88 million by 2050^4^, it is increasingly important to study EM. EM is the remembrance of past personal events and experiences, and it is supported by a distributed network of cortical and subcortical brain structures, including the involvement of the hippocampus and the prefrontal cortex^1,5,6^. A decline in EM is associated with a decrease in the ability to perform activities of daily living and an increase in social isolation^7,8^. Memory complaints often begin to occur even in midlife, with about one in nine adults ≥45 years reporting memory problems^9^. EM declines early in the aging trajectory because the hippocampus is more susceptible to age-related deterioration than other brain regions^10^. Greater hippocampal volume is important because reduced hippocampal volume is associated with conversion to AD over a 2.4-year follow-up among older adults with mild cognitive impairment (MCI), the transitional phase between normal cognition (NC) and dementia, and among older adults with subjective cognitive decline (SCD; i.e., self-reported memory problems)^11,12^. Given a lack of pharmaceutical treatments to prevent or reverse this deterioration, it is important to investigate non-pharmaceutical methods for promoting the integrity of the hippocampus and EM with increasing age.

Fortunately, participation in aerobic exercise (AE) improves markers of brain health that otherwise decline with age and that are associated with an increased risk for AD^13^. AE is a form of physical activity that is structured, planned, repetitive, and performed at an intensity that maintains or improves cardiorespiratory fitness^14^. Cross-sectional, longitudinal, and randomized controlled trials (RCTs) in older adults demonstrate that AE increases hippocampal gray matter volume^15^, hippocampal blood volume^16^, and hippocampal functional connectivity^17^. AE also strengthens functional connectivity of the hippocampus to the default mode network, a hippocampal-cortical brain network that spans the medial and lateral surfaces of the prefrontal, parietal, and temporal cortices^17^. Further, the default network shows reduced functional connectivity with aging and predicts later cognitive impairment^18,19^. These data suggest that regular exercise may be a low-cost, scalable, and highly accessible treatment to either enhance hippocampal-related function or prevent/delay the risk for hippocampal degeneration typical of aging and early stages of pathological cognitive decline.

Despite the distinct benefits of AE on the hippocampus^20^, it remains unclear whether AE induces changes in the cognitive functions supported by the hippocampus, namely EM. Although rodent studies provide irrefutable evidence that AE enhances hippocampal-related memory task performance, the results are more equivocal in humans^21,22^. While some RCTs have found significant improvements in EM among older adults with and without cognitive impairment^23–25^, others have failed to find an effect^26,27^. Several meta-analyses have summarized the findings from these RCTs and found no benefits of AE on EM among older adults with or without cognitive decline^28–33^.

Several limitations of the aforementioned meta-analyses qualify and complicate the interpretation of these null results. First, prior meta-analyses used restricted inclusion criteria, limiting the analyses to studies of either only individuals with NC or only individuals with MCI. By examining each group in isolation, we may miss important information about the trajectory of cognitive aging that would help determine when exercise could be most beneficial. Second, the aforementioned reviews offer little information about the optimal dose of AE, such as intervention length, for positively influencing EM in particular. Determining the doses necessary for improving EM has both theoretical and practical value, as it could help inform mechanistic explanations as well as future interventions and health professionals about levels of AE to prescribe. Additionally, sample characteristics, such as sex distribution, were often overlooked in previous analyses. Numerous RCTs have shown that AE elicits greater cognitive benefits in females than in males^34,35^. Further, females tend to outperform males on tests of EM, and males experience greater incidence rates of EM decline than females^36,37^. Thus, pooling studies with different sample and intervention characteristics may increase heterogeneity and mask significant effects. Third, some of the aforementioned reviews examine the broader domain of memory, which often includes working memory tasks and other tasks not specific to EM. Lastly, 15 relevant studies have been published since the last meta-analysis^30^. Consequently, there is a need for a more complete summary of the available evidence of older adults without dementia, with a focus on the impact of sample and intervention characteristics.

To address the gaps in previous meta-analyses, the current meta-analysis examined dose parameters as potential moderators of the effect of AE on EM and included studies of *all* adults with a mean age ≥55 years without dementia, encompassing the trajectory of cognitive aging from NC to MCI. Thus, this study addresses the following two aims: (1) examine the effects of AE RCTs on EM in adults ≥55 years without dementia; and (2) assess whether any of the following characteristics of the sample and intervention moderate the effect of AE on EM: sex distribution, cognitive status, age, control group type, reporting of prescribed intensity, intervention length, session duration, session frequency, and intervention volume. We predicted that AE would improve EM and that this effect would be greater in NC, females, younger ages, interventions with a no-contact control group, and interventions with greater amounts of prescribed exercise. Our results show that AE positively influences EM, with larger effects observed among various sample and intervention characteristics. These results have far-reaching clinical and public health relevance, highlighting the importance of implementing AE early on in the aging process to help mitigate deficits in EM apparent in healthy aging, AD, and other neurodegenerative diseases, such as Parkinson’s disease and Huntington’s disease.

## Methods

We conducted this meta-analysis in accordance with established guidelines from Preferred Reporting Items for Systematic Reviews and Meta-Analysis (PRISMA)^38^. Before conducting the search, we registered the proposed meta-analysis on PROSPERO (Registration number: CRD42020222666).

### Inclusion and exclusion criteria

Studies were included if they met the following criteria (see Table 1): (1) Studies of males and/or females with a mean age of 55 years or older. All studies of participants with NC, SCD, and MCI were included, regardless of the diagnostic criteria used. Studies that included clinical samples with other neurological or mental illnesses (e.g., dementia, stroke, depression) were excluded; (2) RCTs of AE (including aerobic dance and aerobic tai chi interventions). Studies that included strength training, exergaming, or cognitive training as part of the AE group were excluded because of their potential confounding impact on cognition through nonaerobic mechanisms. One study that produced three publications described their methods as including some sessions in the anaerobic zone^39,40^. These publications were included here because the majority of the duration of the intervention was conducted in the aerobic zone; (3) Control group type could include no contact, wait-list, stretching, toning, balance, light resistance, education, or social interaction. Studies with strength training, exergaming, or cognitive training in the control group were excluded; (4) EM measure consisting of word list, story recall, face/name recognition, object memory, or paired associates. Studies using only composite scores were excluded if the composite score included a measure not satisfying the EM criteria above; and (5) Study design of any length, frequency, duration, volume, or intensity, but acute exercise interventions were excluded. Study design must have isolated the effects of the aerobic group from the control group.

**Table 1:** Inclusion criteria.

Parameter	Inclusion criteria	Exclusion criteria
Participants	Adults with a mean age of 55 years or older with normal cognition, subjective cognitive decline, or mild cognitive impairment	Clinical samples (e.g., depression, dementia)
Intervention	Randomized controlled trial of an aerobic exercise intervention	Strength training, exergaming, or cognitive training in the aerobic group
Control	No contact, wait-list, stretching, toning, balance, education, or social interaction	Strength training, exergaming, or cognitive training
Outcomes	Episodic memory measure, including word list, story recall, face/name recognition, object memory, or paired associates	Composite score that did not exclusively assess episodic memory
Study design	Any length, frequency, duration, volume, or intensity	Did not isolate effects of aerobic group from control group

### Search strategy

**Fig. 1:**
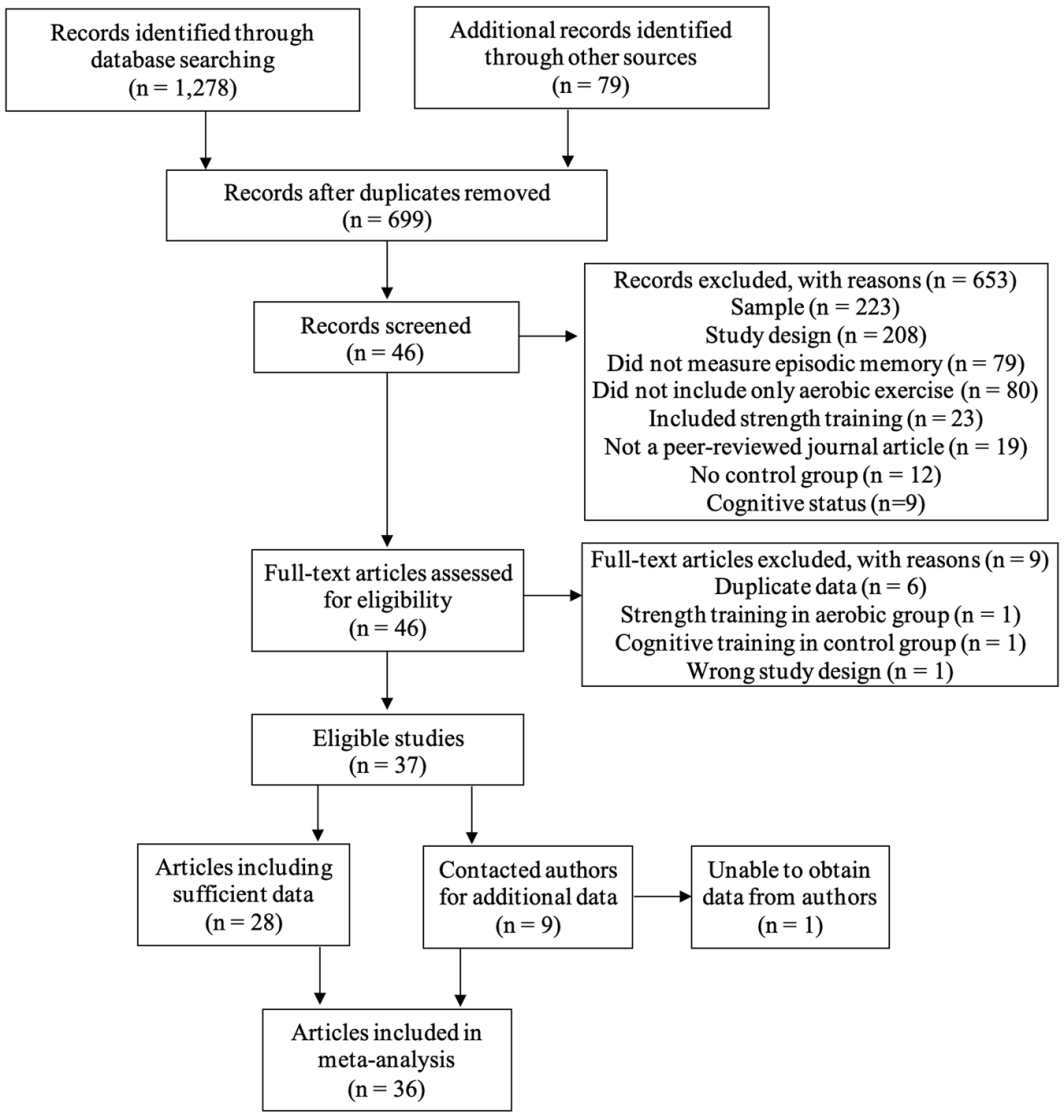
Prisma flow diagram. Adapted from: Moher D, Liberati A, Tetzlaff J, Altman DG, The PRISMA Group (2009). Preferred Reporting Items for Systematic Reviews and Meta-Analyses: The PRISMA Statement. PLoS Med 6(7): e1000097. doi:10.1371/journal.pmed1000097

A literature search was conducted on April 1st, 2021, using the following databases: PubMed, CINAHL, EMBASE, PsycINFO, and CENTRAL. The following combination of search terms was used: memory OR recall OR verbal learning test OR reminding test OR story recall OR list learning OR word list OR paired associations AND randomized control trial* OR clinical trial OR RCT AND exercise* OR physical activity OR physical training AND aerobic AND older adult* OR aging OR aged OR elderly OR geriatric OR cognitive impairment OR cognitive decline OR memory decline (see Supplementary Data 1 for database-specific syntax). We included only English-language articles. All articles published before April 1st, 2021 were included. We identified additional titles by a manual search of relevant journals (International Journal of Sports Medicine, Frontiers in Aging Neuroscience, and British Journal of Sports Medicine) and by identifying references in the six aforementioned meta-analyses. All returned titles were uploaded to Rayyan QCRI, a web application for systematic reviews^41^. The first author screened all returned titles to exclude duplicate studies. The first and second authors independently screened the remaining 699 titles and abstracts for eligibility based on the inclusion criteria described above. The authors agreed on the eligibility decision for 673 articles (96%); the first author resolved disagreements on the remaining 26. If articles appeared to be eligible but did not provide enough data to estimate an effect size or enough information about the intervention, the first author contacted the study’s corresponding author (*k* = 9). Eight of the contacted authors were able to provide the requested data (*k* = 8). One of these studies included both MCI and dementia patients, and the author provided the requested data for only the patients with MCI^42^. Six studies included in previous meta-analyses were excluded from our analysis for the following reasons: (1) did not include a measure primarily evaluating EM^15,43^, (2) EM composite score included a measure that was not primarily evaluating EM^23^, (3) AE group included strength training^24,44^, and 4) did not include a control group^45^. In sum, 36 studies measured EM following an AE intervention and were included in the meta-analysis (Fig. 1).

### Data extraction

All data were extracted and coded by the first author (see Supplementary Table 1 for a full list of variables and Supplementary Data 2 for the source data underlying all analyses, figures, and tables). All extracted variables were reviewed by the second author to ensure data accuracy. Where available, the pre and post mean, the pre and post standard deviation, and the sample size for the exercise and control groups at baseline (a conservative intent-to-treat framework) were considered in the calculation of effect size. When these data were not available, change from baseline, the standard deviation of the mean change, and the number of participants at each assessment for the exercise and control groups was extracted. Consistent with previous meta-analyses^30^, we assumed a moderate (0.5) correlation between baseline and post-intervention values. Improvement in EM performance was coded as a positive change score.

### Data coding

#### Sample characteristics

Sex distribution, cognitive status, and age were coded into categorical variables. Sex distribution was coded using a median split: low (0–64% female) or high (65–100% female). Cognitive status was coded into one of two categories—normal or impaired—using the study-defined diagnosis. Mean age was coded using a median split: young-old (55–68 years old) or old-old (69–85 years old). For a list of studies and their coded variables of interest, see Supplementary Data 3.

#### Intervention characteristics

Control group type, reporting of prescribed intensity, intervention length, session duration, session frequency, and intervention volume were coded into categorical or ordinal variables. The possibility of finding effects of an AE intervention relative to control is likely influenced by features of the control group. Thus, we coded control group type into three categories: no-contact (standard medical practice or wait-list), inactive (inactive control condition not involving any form of physical activity, such as education, painting, and social interaction), or physically active (involving physical activity without an aerobic component, such as stretching). Due to the infrequency of studies adequately reporting the prescribed intensity of the AE intervention, we categorized prescribed intensity simply as reported or not reported. Intervention length was coded as number of weeks and categorized into tertiles based on the distribution of included studies: short (6–17 weeks), medium (18–39 weeks), or long (40–65 weeks). When intervention length was reported in months, a conversion to weeks was calculated using the following criteria: 3 months = 13 weeks, 4 months = 17 weeks, 6 months=26 weeks, and 12 months = 52 weeks. Session duration was coded as the maximum number of minutes per exercise session, including any time spent warming up and cooling down. Session duration was coded using a median split: short (15–45 min) or long (50–90 min). Session frequency was coded into tertiles based on the distribution of included studies: low (1–2 sessions/week), medium (3 sessions/week), and high (4–7 sessions/week). When a study required a certain number of in-person supervised sessions/week and encouraged participants to exercise at home during the week, the number of in-person supervised sessions was coded. Intervention volume was operationalized as total training minutes and calculated as the product of the intervention length, frequency, and duration. Intervention volume was coded into tertiles based on the distribution of included studies: low (<2100 total minutes), medium (2100–3900 total minutes), or high (>3900 total minutes).

### Quality assessment

The Physiotherapy Evidence Database (PEDro) scale was used as a template to assess study quality (Supplementary Table 2)^46^. This scale has been used extensively in the evaluation of methodology of similar studies, and it is a valid and reliable quality assessment tool^47–50^. Study quality was calculated by adding the total number of criteria met on the checklist (possible range = 0–11). The first author assessed the quality of each study, and the second author reviewed the scores to ensure accuracy. Although PEDro does not provide specific instructions for classifying studies, the following criteria were used in accordance with previous meta-analyses:^50,51^ scores 0–3 indicate poor-quality, scores 4–5 indicate fair-quality, and scores 6–11 indicate good- to excellent-quality.

### Analyses

Comprehensive Meta-Analysis software (CMA; Version 3) was used to transform the standard mean difference into Hedges’ *g* and to calculate the variance and 95% confidence interval (CI) for each study’s effect size^52^. In studies with multiple treatment groups, we extracted data only for AE or control groups, excluding other intervention groups, such as those with cognitive training or resistance training. The individual effect sizes were then combined to create a summary effect size using the inverse variance of the individual effect size as weights. Therefore, studies did not contribute equally to the summary effect size; rather, studies with greater variance (less precision) in their effect size estimate contributed less to the summary effect size than studies with smaller variance (greater precision). The summary effect size was calculated using a random-effects model, which assumes that each study has a different true effect size; we expected different true effect sizes for each study since studies were not matched on sample characteristics (e.g., age, cognitive status) or study design (e.g., control group type, dose-parameters, adherence) that could influence the magnitude of the effect size^52^.

For Aim 1, CMA was used to calculate the overall mean effect size of AE on EM by averaging across all measures of EM. Some studies reported means for multiple measures of EM. In these instances, we averaged the effect sizes across the measures and components when estimating the mean effect size for that study. Two studies produced two publications each that examined different measures of EM that were combined for the purpose of this analysis^25,53–55^. In addition, one study produced three publications examining overlapping and different measures of EM using different subsamples; the overlapping measure was included once using the largest subsample and the remaining measures were included as separate studies for the purpose of this analysis^39,40,56^. A leave-one-out analysis was performed, where each study was left out sequentially, to address whether a specific study was influential in the effects of AE on EM. Supplementary analyses were performed to calculate the overall mean effect size of AE on memory scores (total recall across learning trials, immediate recall, delayed recall, or recognition) and task performance (word list or story recall).

We tested for heterogeneity in the effect size by calculating the *Q*-statistic. A *Q*-statistic with a *p* value < 0.05 suggests significant heterogeneity in effect sizes. Significant heterogeneity indicates that the true effect size differs between studies. If significant heterogeneity was detected, an *I*^*2*^ statistic was then calculated to estimate the percentage of the total variability in effect sizes across studies due to true heterogeneity.

For Aim 2, we used CMA to conduct mixed-effects subgroup analyses and meta-regression analyses to investigate the study-specific variables that might moderate the strength of the effect size across studies. Moderation effects were analyzed using the following categorical moderators: sex distribution, cognitive status, age, control group type, reported prescribed intensity, intervention length, session duration, session frequency, and intervention volume. First, the mixed-effects model was used to estimate the within-subgroup effect sizes for each moderator. Then, moderating effects were tested using *Q*-statistics to test the heterogeneity between effect sizes of subgroups defined by each moderator. The *R*^2^ statistic, which denotes the proportion of the true variance explained by the moderator, was obtained from the meta-regression model as the effect size of the moderator. Finally, the estimation of Hedges’ *g* and its corresponding test for the zero effect were conducted stratified by subgroup. All analyses were conducted at the significance level of *p* < 0.05.

#### Sensitivity analyses

We performed a sensitivity analysis to investigate the impact of study quality. It was expected that higher-quality studies would provide more accurate effect size estimates. Therefore, we re-ran the primary analysis to examine whether any association changed when only high-quality studies were included (study quality score ≥ 6). If meta-analytic findings were primarily driven by poorer-quality studies, then we would be more cautious when interpreting results. A sensitivity analysis was also performed for sex distribution using a cut-off of 50% (<50% female or ≥50% female). Sensitivity analyses were also performed to assess whether a pre-post correlation value of 0 or 0.9 impacted the results. A sensitivity analysis was also performed to assess whether removing one publication with a mean age less than 60 years impacted the results^57^. Lastly, a sensitivity analysis was conducted to assess whether removing the three publications that included sessions in the anaerobic zone impacted the results^39,40,56^.

#### Risk of bias

Publication bias was assessed by a funnel plot, Duval and Tweedie’s Trim and Fill, Egger’s unweighted regression asymmetry test, and the Copas selection method. Trim and Fill uses an iterative process to remove the most extreme studies with small sample sizes, recompute an unbiased estimate of the effect size, and create a funnel plot that includes the observed studies and the imputed studies. If the shift of the effect size is small, we can be more confident that the reported effect is valid. A *p* value of Egger’s asymmetry test > 0.05 indicates a symmetrical distribution of effect sizes and a low risk of publication bias. The Copas selection model gradually increases the positive association between the precision of a study and the probability that it is included in the meta-analysis until the test for residual selection bias is nonsignificant^58,59^. The Copas selection model was conducted at the significance level of 0.1 to test for residual selection bias using the R package metasens^60^.

#### Statistical power

A retrospective power calculation was conducted for the moderating effects using the *power.analysis.subgroup* function from the R package dmetar^61^. We also computed the sample size required for a future RCT to achieve 80% power for a two-sided *t*-test at the significance level of *p* = 0.05.

## Results

### Study characteristics

The initial search yielded a total of 1278 potentially relevant studies (Fig. 1). Of these, 36 RCTs met all inclusion criteria, representing data from 2750 participants (see Supplementary Data 4 for a list of studies). All included studies provided information about our moderators of interest and were published between April 1985-March 2021. Study sample sizes across exercise and control groups ranged from 15–389 participants, with a mean sample size per group per study of 38. The average age ranged from 59 to 85 years, with a sample-size-weighted mean age of 70.82 years. Mean age was similar across exercise (70.64) and control groups (71.01). Females comprised 66.40% of the participants across all studies. Twenty studies included individuals with NC and 16 studies included individuals with impaired cognition, consisting of 1 SCD and 15 MCI studies. One study involved a placebo in both the aerobic and control groups^35^.

### Effects of aerobic exercise on episodic memory

**Fig. 2:**
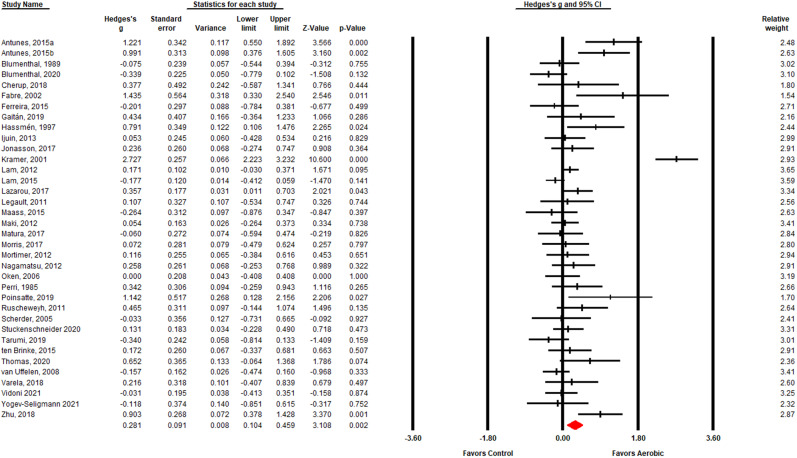
Forest plot of individual studies (*n* = 36) and pooled effects of aerobic exercise interventions on episodic memory. Hedges’ *g*, denoted as a diamond or a square, reflect improvements in episodic memory among those in the aerobic exercise group relative to the control group when the value is positive. A random-effects model was used. Error bars stand for 95% CI.

Figure 2 presents a summary of the effects of AE on EM using data from 36 RCTs. Consistent with our hypothesis, we found that AE enhanced EM (*g* [95% CI] = 0.28 [0.10–0.46]; *p* = 0.002). Given the observed effect size and CI, 404 participants (202 per intervention group) are needed in future studies to achieve 80% statistical power. Sensitivity analyses revealed similar results when a pre-post correlation value of 0 (*p* = 0.005) or 0.9 (*p* < 0.001) was used. Sensitivity analyses revealed similar results when excluding the three publications that included anaerobic sessions. Sensitivity analyses also revealed similar results when excluding one publication with a mean age less than 60 years. When removing each study in turn with a leave-one-out cross-validation analysis, all *g*’s remained >0.17. and all *p*’s remained < 0.005. Supplementary analyses revealed a significant positive effect of AE on all memory scores, except learning and recognition, and all tasks (Supplementary Figs. 1–6).

**Fig. 3:**
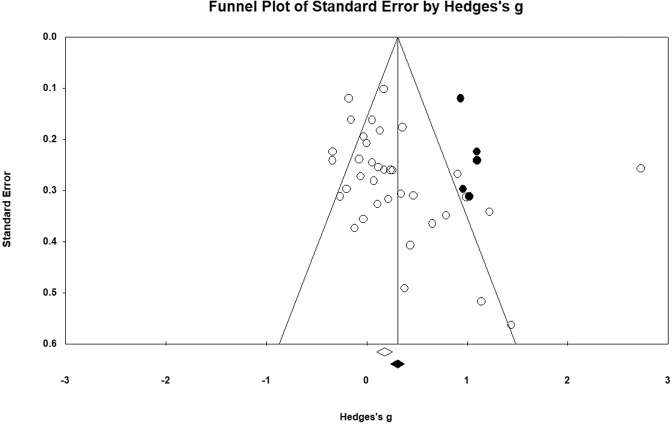
Funnel plot of standard error by Hedges’ g with imputed studies. The observed studies (*n* = 36) are shown as open circles and the observed Hedges’ g is shown as an open diamond at 0.28 [0.10–0.46]. The five imputed studies are shown as filled circles and the imputed Hedges’ g is shown as a filled diamond at 0.38 [0.20–0.56].

We found significant heterogeneity in the full sample (*Q*(*df*) = 170.00 (35); *p* < 0.001), such that 79.41% of the variation in effect sizes could be attributed to true differences in the effect size across studies. Egger’s unweighted regression asymmetry test suggests potential publication bias (*t*(34) = 2.07; *p* = 0.023). The Trim and Fill analysis suggests that if we remove the asymmetric studies, the effect size would increase to *g* [95% CI] = 0.38 [0.20–0.56] (Fig. 3). Using the Copas selection model, the adjusted effect size is smaller than the original effect size and no longer statistically significant (g [95% CI] = 0.10[−0.04, 0.24]; *p* = 0.16). Further, the Copas selection model revealed an estimated probability of publishing the trial with the largest standard error of 0.71 and an approximated number of unpublished studies of 14.

### Moderation analyses

We tested whether the following sample and intervention characteristics moderated the strength of the effect size across studies: sex distribution, cognitive status, age, control group type, reported prescribed intensity, intervention length, session duration, session frequency, or intervention volume (Table 2). The results of the meta-regression analysis suggested that only age acted as a significant moderator (*Q*-statistic = 4.92; *p* = 0.027). The effect size was moderately small (*R*^2^ = 0.09). The effect sizes for the other moderators were small and did not provide statistical evidence of a moderating effect at *p* < 0.05.

**Table 2:** Moderation Analyses.

Moderator	No. of studies	Hedges’ g	Standard error	Lower limit	Upper limit	*p*-value	*Q*-statistic	*p*-value^†^	*R*^2^
Sex Distribution (percent)							0.34	0.558	<0.01
Low (0–64)	18	0.23	0.14	−0.03	0.5	0.086			
High (65–100)	18	0.34	0.13	0.09	0.6	0.009			
All	36	0.29	0.09	0.1	0.47	0.002			
Cognitive Status							2.43	0.119	0.04
Impaired	16	0.14	0.13	−0.12	0.39	0.3			
Normal	20	0.42	0.13	0.17	0.66	0.001			
All	36	0.29	0.09	0.11	0.46	0.002			
Age (years)							4.92	0.027	0.09
Young-Old (55–68)	18	0.49	0.13	0.24	0.75	<.001			
Old-Old (69–75)	18	0.1	0.12	−0.14	0.34	0.418			
All	36	0.28	0.09	0.11	0.46	0.001			
Control Group Type							1.55	0.461	<0.01
No-Contact	12	0.38	0.16	0.07	0.7	0.018			
Inactive	12	0.12	0.16	−0.20	0.44	0.456			
Physically Active	12	0.35	0.16	0.04	0.67	0.028			
All	36	0.29	0.09	0.11	0.47	0.002			
Intensity Reporting							1.59	0.208	<0.01
Not Reported	9	0.1	0.18	−0.25	0.45	0.587			
Reported	27	0.36	0.11	0.15	0.57	0.001			
All	36	0.29	0.09	0.1	0.47	0.002			
Intervention Length (weeks)							2.3	0.316	0.01
Short (6–17)	12	0.25	0.17	−0.08	0.57	0.14			
Medium (18–39)	13	0.46	0.15	0.16	0.76	0.002			
Long (40–65)	11	0.13	0.16	−0.18	0.44	0.405			
All	36	0.29	0.09	0.11	0.47	0.002			
Session Duration (min)							0.09	0.76	<0.01
Short (15–45)	17	0.32	0.14	0.05	0.59	0.021			
Long (50–90)	19	0.26	0.13	0.01	0.51	0.04			
All	36	0.29	0.09	0.1	0.47	0.002			
Session Frequency (sessions/week)							1.58	0.453	<0.01
Low (1-2)	11	0.14	0.16	−0.18	0.45	0.387			
Medium (3)	20	0.39	0.13	0.14	0.64	0.002			
High (4-7)	5	0.23	0.26	−0.27	0.74	0.369			
All	36	0.29	0.09	0.11	0.47	0.002			
Intervention Volume (total min)							0.19	0.909	<0.01
Low (<2100)	12	0.23	0.17	−0.11	0.57	0.188			
Medium (2100–3900)	12	0.31	0.16	−0.01	0.63	0.06			
High (>3900)	12	0.33	0.17	0.004	0.65	0.047			
All	36	0.29	0.1	0.1	0.48	0.003			

^†^*p*-value of the heterogeneity between effect sizes of subgroups.

Mixed-effects subgroup analyses revealed a significant effect for studies with a high percentage of females (*g* = 0.34; *p* = 0.009) but not those with a low percentage of females (*g* = 0.23; *p* = 0.086). Similar results were found when using 50% as the cut-off. These subgroup analyses also indicated a significant effect for NC (*g* = 0.42; *p* = 0.001) but not for participants with impaired cognition (*g* = 0.14; *p* = 0.300). In line with the moderator analyses described above, there was a significant effect for studies with young-old participants (*g* = 0.49; *p* < 0.001) but not for old-old participants (*g* = 0.10; *p* = 0.418). The type of control group also influenced the results; there was a difference in EM when studies used a no-contact (*g* = 0.38; *p* = 0.018) or physically active (i.e., stretching) (*g* = 0.35; *p* = 0.028) control group but not when studies used an inactive control group (i.e., education, social interaction) (*g* = 0.12; *p* = 0.456). Interestingly, the effect of AE on EM was significant for studies that reported their prescribed intensity (*g* = 0.36; *p* = 0.001) and not for studies that did not report prescribed intensity (*g* = 0.10; *p* = 0.587). Features of the intervention also appeared to influence the effects. Specifically, there was a significant effect for studies of medium length (*g* = 0.46; *p* = 0.002) but not for short (*g* = 0.25; *p* = 0.140) or long (*g* = 0.13; *p* = 0.405) interventions. There was an effect for studies with both short (*g* = 0.32; *p* = 0.021) and long (*g* = 0.26; *p* = 0.040) session durations. Studies with medium frequency (*g* = 0.39; *p* = 0.002) had a significant effect, but studies with low (*g* = 0.14; *p* = 0.387) or high (*g* = 0.23; *p* = 0.369) session frequency did not. Finally, there was a significant effect for studies with a high volume of prescribed AE (*g* = 0.33; *p* = 0.047) but not for studies with a low (*g* = 0.23; *p* = 0.188) or medium (*g* = 0.31; *p* = 0.060) volume.

### Quality assessment

Twenty-eight of the 36 studies were judged to be of high methodological quality because they scored ≥6 on the 11-item quality criteria. Supplementary Data 3 presents details on the methodological quality of each study. A post hoc analysis revealed a significant positive effect of AE on EM when only high-quality studies were included (*g* [95% CI] = 0.18 [0.03-0.32]; *p* = 0.016).

## Discussion

In accordance with Aim 1, our meta-analysis of adults ≥55 years without dementia demonstrates that participation in AE improves EM. In accordance with Aim 2, we found that AE improved EM in studies with NC, those with a mean age of 55–68, and those above the median in female participants. We also found that the EM exercise effect was only significant in studies with a physically active or no-contact control group, that reported a prescribed AE intensity, and with session durations lasting 15–90 min three sessions/week for 18–39 weeks to achieve >3900 total minutes of activity. These observations have high practical relevance for the use of AE as a low-cost, accessible, non-pharmaceutical intervention to improve EM in late adulthood.

Previous meta-analyses had reported that AE does not influence EM among older adults with and without cognitive impairment^28–32^. The discrepancy in findings between this meta-analysis and previous meta-analyses may be due to methodological differences. Prior meta-analyses had restricted inclusion criteria, leading to a smaller number of included studies with AE and EM outcomes (e.g., *N*_Northey_ = 9). We included all studies of participants without dementia, which permitted us to include an additional 20 RCTs not included in previous meta-analyses. Further, another strength of this meta-analysis is the strict EM inclusion criteria, excluding studies with a measure not primarily targeting EM, such as spatial working memory, that was previously included in meta-analyses. By collapsing across various EM tasks that exhibit differential task demands on the hippocampus or recruit heterogeneous anatomical correlates, previous meta-analyses may have reduced their sensitivity to detect an effect.

Many studies included in the analyses did not reveal significant AE effects when considered individually, likely due to insufficient power because of small sample sizes. The power analysis revealed that 404 participants (202 per group) are needed in future studies to achieve 80% statistical power. None of the studies included in the meta-analysis had a sample size of this magnitude; the largest sample size was 389^62^, which gave 78.75% power to detect an effect size of *g* = 0.28, but studies on average had only 38 participants per group. The evidence for an AE effect became much stronger when all the data were pooled since most effects were in the positive direction.

Supplementary analyses revealed a significant positive effect of AE on immediate recall and delayed recall scores but not learning or recognition scores. There is research to suggest that conventional memory networks may not be sufficient or important for total learning performance and interpreting learning scores as episodic memory may be inaccurate^63^. These results are also consistent with the long-standing finding in cognitive psychology that age differences are smaller for recognition than recall^64^, such that there may be fewer age-related deficits that need to be mitigated. The literature suggests that these age differences in recall and recognition may be due to a common underlying process rather than ceiling effects in recognition tasks, such that older adults rely more on a general feeling of familiarity and are impaired in recollecting specific events, which is more important in recall tasks^64^. Further, the underlying process of familiarity is thought to rely on a more diffuse network of cortical and subcortical regions than the underlying process of recollection, which is closely linked with the hippocampus^65^. Thus, these regions associated with familiarity may be less modifiable with AE. It cannot be ruled out that the type of recognition score (i.e., hits, false alarms, d’, percent accuracy) could have influenced the results, as most studies used percent accuracy or did not specify the measure used. We also found a significant positive effect of AE on word-list and story-recall tasks. Due to an insufficient number of studies examining face/name recognition, object memory, or paired associates, we were not able to examine or compare the effect of AE on these dimensions of EM. We were also not able to examine differences between studies that assess the “what,” “where,” or “when” components of EM given that most studies employed word list recall (75%) or story recall (33%) tasks, which tap the “what” component. We did not include spatial working memory or constructional praxis tasks due to their recruitment of heterogeneous anatomical correlates outside of the hippocampus. Thus, we were not able to distinguish effects on more distinct aspects of EM, such as visuospatial memory, or compare verbal memory to visuospatial memory. Future studies would benefit from comparing verbal memory with visuospatial memory to help provide information about the specificity of AE effects.

We had sufficient power to detect moderating effects of age, so any other possible moderators should be interpreted with caution. Moderation analyses revealed that age was a significant moderator of the effects of AE on EM. One explanation for our results is that there may be an opportune window during which AE has a more profound effect on EM. In particular, the larger benefits of AE for the young-old group may result from age differences in general health; comorbid conditions, medications, safety concerns, and other neuropathological issues, which are often more prevalent in older populations, could negatively impact adherence or the efficacy of the intervention. However, our results do not allow us to determine whether AE earlier in the lifespan can change the trajectory of cognitive aging because the current meta-analysis focused on adults with a mean age ≥55 years at baseline. To address this gap, future studies would benefit from examining a younger population and following them across time to assess how AE at a single time point impacts EM later in life.

Although we did not find significant moderation as a function of sex distribution, subgroup analyses demonstrated that AE had favorable effects among studies composed mostly of female participants. These sex effects may be driven by differences in encoding strategies, such that females tend to utilize strategies (i.e., semantic clustering, word rhymes) that are associated with better performance^66^. It is possible that AE enhances the utilization or effectiveness of these strategies during encoding. These sex effects may also be driven by differences in exercise efficacy. Numerous studies have shown greater effects of AE for females compared to males on measures of executive functioning and information processing speed^34,35^. The sex differences may be related to sex steroid hormones estradiol and testosterone^29^. Extensive literature supports the role of sex hormones in neuroplasticity and the preservation of cognitive function, with evidence suggesting that greater circulating levels of estradiol and testosterone after the onset of menopause and andropause are associated with better EM in females but not males^67,68^. During development and reproductive years, the hormonal environment has lasting effects on brain structure, brain function, muscles, adipose tissue, and other organs^69,70^. Further, acute and chronic exercise increase sex steroid levels in the brain, muscles, and circulation^71^. Thus, even in late adulthood, exercise influences the brain and organs in a sex-dependent manner.

Subgroup analyses demonstrated that AE had favorable effects among participants with NC but not among participants with impaired cognition. There are several potential reasons for null results in impaired populations. Adherence to interventions varies greatly among studies of patients with MCI due to practical, psychological, and cognitive factors^72^. Additionally, given that our categorization of normal and impaired was based on study-defined diagnoses, there was significant heterogeneity across studies regarding diagnostic criteria—for example, one study^73^ defined normal cognition as ≥24 on the Mini-Mental State Examination (MMSE), while another study used the same criteria to define MCI^35^, and yet another study defined MCI as a score of 7 or higher on the 12-item version of the MMSE^27^. This variability in diagnostic criteria may have hindered our ability to detect a significant effect among patients with impaired cognition. Lastly, although higher levels of physical activity are associated with reduced disease progression in patients with MCI^74^, the magnitude of the benefits may be smaller among individuals who have already developed neuropathology. Our results suggest that AE may be maximally beneficial among adults ≥55 years who are sedentary before any changes in cognition are detected clinically.

Although control group type was not a significant moderator, subgroup analyses demonstrated that AE had favorable effects among studies with a no-contact or physically active control group (i.e., stretching) but not among studies with an inactive control group (i.e., education). These finding suggest that while physical activity without a primary aerobic component is insufficient to promote significant changes in EM, education and social interactions may have a marginal effect, and, thus, may dampen the ability to detect significant effects of AE. Education or social interaction control groups may have promoted changes in lifestyle habits that had measurable benefits on EM. However, future research is needed to further explore potential underlying mechanisms.

While prescribed intensity was not a significant moderator, subgroup analyses demonstrated that AE had favorable effects only among studies that reported prescribed intervention intensity. Studies that report prescribed intensity may be better controlled, such that participants are monitored to ensure the target intensity is reached. It is also possible that studies that report prescribed intensity individualize the prescription for each participant based on their pre-training aerobic capacity. Given the heterogeneity across studies in reporting prescribed intervention intensity and the lack of variability among studies that reported prescribed intensity, we were not able to examine whether variation in intensity was a significant moderator of the effects of AE on EM.

Although intervention length, duration, frequency, and volume were not significant moderators, subgroup analyses demonstrated that AE had favorable effects among studies 18–39 weeks long with three sessions/week lasting 15–90 min each, achieving >3900 total minutes of activity. Given these results, the public health recommended target of 150 min/week of moderate-intensity physical activity^75^ can be reached in a variety of ways. For example, 3900 min of activity could include 50-minute sessions 3 days a week for 26 weeks. Our results show that interventions 6–17 or 40–65 weeks, interventions with 1–2 or 4–7 sessions/week, and interventions prescribing ≤3900 total training minutes were not robust enough to result in significant differences in EM. It is possible that the most intense interventions—those 40–65 weeks or 4–7 sessions/week – yielded null results because interventions with greater time commitments could have had several limitations minimizing their observed effects, including greater subject burden, decreased adherence, increased attrition and withdrawal, and less precise measurement of exercise adoption. However, given the heterogeneity across studies regarding reporting these values, we could not examine whether variation in rates of adherence or attrition significantly moderated the effects described here. Further, intervention length and frequency varied substantially even among studies with >3900 total minutes of activity; interventions ranged from 26 to 65 weeks long with 2–7 sessions/week. This limits our ability to definitively state an optimal exercise dose at which EM benefits are detected or strengthened. Nonetheless, our results indicate that the public health recommendations of 150 min/week would require interventions greater than 26 weeks to detect EM improvements among adults ≥55 years.

There are likely many behavioral and cellular mechanisms by which AE influences EM. For example, moderate-intensity exercise interventions improve sleep quality, which has a critical role in memory consolidation^76,77^. Exercise also improves mood, which can positively impact memory, although direct tests of these possible mechanisms from RCTs remain inconclusive^78–80^. There are also many physiological changes following exercise, including decreased body weight^81^ and cardiovascular responses and reactivity^82^, which could influence brain health outcomes, including EM. EM is dependent on hippocampal function^83^, and exercise appears to positively impact hippocampal structure and function, especially in older adult populations^15–17^. On a cellular level, AE increases the expression and secretion of numerous growth factors and neurotrophins in the hippocampus, such as brain-derived neurotrophic factor (BDNF), which in turn enhances synaptic plasticity, cell proliferation, and survival^84–86^. In humans, an AE-related increase in hippocampal volume was associated with increased circulating levels of BDNF^15^. Additionally, AE might regulate an anti-inflammatory response in humans by modulating anti-inflammatory and proinflammatory cytokines^87^, and in rats, this change in cytokine levels in the hippocampus reduced hippocampal-related memory decline^88^. However, the behavioral and cellular transducers of the benefits of AE in humans are not fully understood and should be further investigated to optimally prescribe therapeutic interventions.

This literature is marked by several methodological limitations. The limited ethnic and racial diversity across samples restricts the generalizability of these results. Further, intervention intensity was often not reported and limited in variability, so a more sensitive classification could not be used in this review. The included studies also did not consistently report supervision, change in cardiorespiratory fitness, withdrawal/attrition, and attendance/adherence, limiting our ability to systematically examine whether these key intervention characteristics impact the effect of AE on EM. Future studies would benefit from reporting these aspects of the fidelity of the intervention so meta-analyses can clearly summarize the impact of these measures. Finally, studies varied in the way missing data was handled, with some studies using an intent-to-treat approach and others using a per-protocol approach depending on the purpose of the study. Studies using a per-protocol approach may have produced biases that may have overestimated the effect. In addition, there is evidence of publication bias and asymmetry; while the more conservative Copas selection model revealed a smaller effect size after accounting for the effects of small studies, the Trim and Fill suggests an effect size significantly larger than 0. Further, although we followed the PRISMA procedure for risk of bias, there are limitations of PRISMA and the PEDro scale that could influence the results from this meta-analysis (e.g., the summary score and cutoff do not acknowledge that some criterions are more critical to the validity of a study than others). Many of the included studies did not report EM performance for males and females separately or young-old and old-old participants separately, requiring us to categorize each study into one of two subgroups. However, future studies would benefit from reporting the effect for each group separately (e.g., males versus females) to allow for a more appropriate examination of these moderators using effect sizes for each group. In addition, we did not have access to individual participant data, which would have allowed for a more complex analysis of the impact of the moderators on EM. However, evidence suggests that group analyses are adequate and even outperform individual analyses in situations with a small number of trials per condition^89^. Further, even individual-level data are essentially grouped data, as they are aggregated across multiple stimulus items^90^. Given that many of the moderators had small effect sizes, future research is needed to further validate the trends that appeared in these data, as well as other moderators that may be important.

Future research would also benefit from more long-term follow-up after the completion of the intervention to assess whether the benefits persist and whether they could delay progression to MCI or dementia. In addition, future studies must examine which type of training can be prescribed to promote the greatest benefits on EM. This may include multimodal training or resistance training, supervised or unsupervised training, group or individual training, and low-, moderate- or high-intensity training. Finally, while the effect of AE on EM was small and required a large sample size to detect a significant effect, a greater focus on middle-aged adults would allow for an investigation of whether benefits of AE on EM are more observable before the age of 55.

AE positively influences EM among adults ≥55 years without dementia, with larger effects observed among various sample and intervention characteristics – the clearest moderator being age. These results suggest that EM should be routinely monitored starting at age 55 or earlier so that interventions can be implemented before changes in cognition become clinically apparent. The subgroup results could inform practitioners about how AE can maximally benefit EM in this age range. These findings highlight the importance for health professionals to communicate the many benefits of exercise for brain health before the appearance of memory problems.

### Reporting summary

Further information on research design is available in the Nature Research Reporting Summary linked to this article.

## Supplementary information

Description of Additional Supplementary Files

Supplementary Information

Supplementary Data 1

Supplementary Data 2

Supplementary Data 3

Supplementary Data 4

Reporting Summary

## Data Availability

Source data underlying all analyses, figures, and tables are available in Supplementary Data 2.
