# Supplementary material for: Aerobic exercise improves episodic memory in late adulthood: a systematic review and meta-analysis
Source: Commun Med (Lond). 2022 Feb 17;2:15. doi: 10.1038/s43856-022-00079-7 (PMC9053291; doi:10.1038/s43856-022-00079-7)
Supplement: Supplementary file 1 — Description of Additional Supplementary Files [file 43856_2022_79_MOESM1_ESM.pdf]

Description of Additional Supplementary Files

File Name: Supplementary Data 1

Description: Database-specific syntax

File Name: Supplementary Data 2

Description: Source dataset underlying all analyses, figures, and tables.

File Name: Supplementary Data 3

Description: Randomized controlled trials examining the effect of aerobic exercise on episodic memory. Sample size includes only the intervention and control group. I = Immediate; D = Delayed; L = Learning; R = Recognition; Paired Assoc. = Paired Associates; Pict. Sequence = Picture Sequence; Face Recog. = Face Recognition; Pict. Recog. = Picture Recognition; Object Mem. = Object Memory; Percent Female = Low: 0-64%, High: 65-100%; Age = Young-Old: 55-68years, Old-Old: 69-85 years; Length = Short: 6-17 weeks, Medium: 18-39 weeks, Long: 40-65 weeks; Session Duration = Short: 15-45 minutes, Long: 50-90 minutes; Session Frequency = Low: 1-2 sessions/week, Medium: 3 sessions/week, High: 4-7 sessions/week; Volume = Low: <2,100 minutes, Medium: 2,100-3,900 minutes, High: >3,900 minutes; Quality = Low: <6, High: ≥6.

File Name: Supplementary Data 4

Description: List of included studies
