# Supplementary material for: Aerobic exercise improves episodic memory in late adulthood: a systematic review and meta-analysis
Source: Commun Med (Lond). 2022 Feb 17;2:15. doi: 10.1038/s43856-022-00079-7 (PMC9053291; doi:10.1038/s43856-022-00079-7)
Supplement: Supplementary file 2 — Supplementary Information [file 43856_2022_79_MOESM2_ESM.pdf]

Aerobic Exercise Improves Episodic Memory in Late Adulthood: A systematic review and meta-analysis

Sarah L Aghjayan, MS, Themistokles Bournias, Chaeryon Kang, PhD, Xueping Zhou, MHS, Chelsea M Stillman, PhD, Shannon D Donofry, PhD, Thomas W Kamarck, PhD, Anna L Marsland, PhD, Michelle W Voss, PhD, Scott H Fraundorf, PhD, Kirk I Erickson, PhD

Supplementary Tables

Supplementary Table 1. List of Coded Variables

Article Information	Sample Demographics
Reference	Age (M, SD, young-old, old-old)
Publication year	Sample size (N)
Quality of study	Sex distribution (low, high)
	Cognitive status (normal, impaired)
Intervention Characteristics	
Control group type (no-contact, inactive, physically active)	Episodic Memory
Intensity reporting (reported, not reported)	Task (word list, story recall, face recognition, paired associates, object memory)
Intervention length (short, medium, long)	Condition (learning, immediate-recall, delayed-recall, recognition)
Session duration (short, long)	Pre-intervention score (M, SD)
Session frequency (low, medium, high)	Post-intervention score (M, SD)
Intervention volume (low, medium, high)	Standardized mean difference (M, SD)

Note. M = mean; SD = standard deviation; N = sample size

Supplementary Table 2. PEDro Template Used for Assessing Study Quality

#	Criteria	Score
1	Eligibility criteria were specified	
2	Subjects were randomly allocated to groups	
3	Allocation was concealed	
4	The groups were similar at baseline regarding the most important prognostic indicators	
5	There was blinding of all subjects	
6	There was blinding of all personnel who administered the intervention	
7	There was blinding of all assessors who measured at least one key outcome	
8	Measures of at least one key outcome were obtained from more than 85% of the subjects initially allocated to groups	
9	All subjects for whom outcome measures were available received the treatment or control condition as allocated or, where this was not the case, data for at least one key outcome was analyzed by “intention to treat”	
10	The results of between-group statistical comparisons are reported for at least one key outcome	
11	The study provides both point measures and measures of variability for at least one key outcome	

Supplementary Figures

Study Name	Hedges's g	Standard error	Variance	Lower limit	Upper limit	Z-Value	p-Value	Relative weight
Blumenthal, 1989	-0.006	0.240	0.057	-0.475	0.464	-0.024	0.981	8.98
Blumenthal, 2020	-0.339	0.225	0.050	-0.779	0.102	-1.508	0.132	9.48
Fabre, 2002	2.414	0.637	0.406	1.166	3.663	3.791	0.000	2.46
Gaitán, 2019	0.377	0.406	0.165	-0.419	1.173	0.928	0.353	4.94
Lazarou, 2017	0.256	0.176	0.031	-0.089	0.601	1.456	0.145	11.24
Legault, 2011	-0.067	0.326	0.106	-0.706	0.572	-0.207	0.836	6.55
Morris, 2017	0.150	0.282	0.079	-0.402	0.702	0.533	0.594	7.70
Nagamatsu, 2012	0.184	0.260	0.067	-0.326	0.693	0.707	0.480	8.34
Ruscheweyh, 2011	0.465	0.311	0.097	-0.144	1.074	1.496	0.135	6.92
Scherder, 2005	0.095	0.355	0.126	-0.602	0.791	0.266	0.790	5.89
Tarumi, 2019	0.026	0.238	0.057	-0.441	0.492	0.107	0.915	9.03
van Uffelen, 2008	-0.237	0.162	0.026	-0.554	0.081	-1.461	0.144	11.76
Varela, 2018	0.359	0.319	0.102	-0.266	0.984	1.126	0.260	6.72
	0.133	0.109	0.012	-0.080	0.346	1.224	0.221	

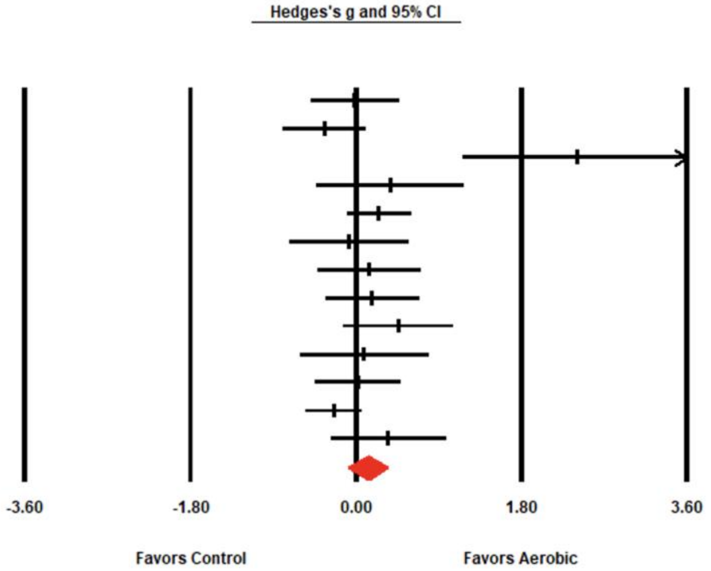

Supplementary Figure 1. Forest Plot of Individual Studies and Pooled Effects of Aerobic Exercise Interventions on Learning

Positive values of Hedges' *g* reflect improvements in learning scores among those in the aerobic exercise group relative to the control group. A random-effects model was used. Results revealed a significant positive effect of aerobic exercise on learning scores.

Study Name	Hedges's g	Standard error	Variance	Lower limit	Upper limit	Z-Value	p-Value	Relative weight
	Statistics for each study							
Antunes, 2015a	1.071	0.336	0.113	0.414	1.729	3.193	0.001	5.09
Blumenthal, 1989	-0.144	0.239	0.057	-0.612	0.325	-0.601	0.548	5.64
Fabre, 2002	0.456	0.480	0.230	-0.484	1.396	0.952	0.341	4.22
Ferreira, 2015	-0.068	0.296	0.088	-0.649	0.512	-0.231	0.817	5.32
Hassmén, 1997	0.134	0.322	0.104	-0.497	0.766	0.416	0.677	5.17
Ijuin, 2013	0.081	0.245	0.060	-0.400	0.562	0.330	0.741	5.61
Jonasson, 2017	0.145	0.260	0.067	-0.363	0.654	0.560	0.575	5.53
Kramer, 2001	2.847	0.260	0.068	2.337	3.356	10.945	0.000	5.53
Lazarou, 2017	0.408	0.177	0.031	0.061	0.755	2.305	0.021	5.94
Legault, 2011	0.282	0.328	0.107	-0.360	0.924	0.862	0.389	5.14
Maass, 2015	-0.321	0.312	0.098	-0.934	0.291	-1.028	0.304	5.23
Matura, 2017	0.166	0.272	0.074	-0.368	0.700	0.609	0.543	5.46
Morris, 2017	-0.008	0.281	0.079	-0.559	0.543	-0.028	0.978	5.41
Mortimer, 2012	0.024	0.255	0.065	-0.475	0.524	0.095	0.924	5.56
Nagamatsu, 2012	0.288	0.261	0.068	-0.223	0.799	1.106	0.269	5.53
Perri, 1985	0.342	0.306	0.094	-0.259	0.943	1.116	0.265	5.27
Poinsatte, 2019	1.142	0.517	0.268	0.128	2.156	2.206	0.027	4.01
Yogev-Seligmann 2021	-0.098	0.374	0.140	-0.830	0.635	-0.262	0.794	4.86
Zhu, 2018	0.903	0.268	0.072	0.378	1.428	3.370	0.001	5.48
	0.395	0.171	0.029	0.061	0.730	2.318	0.020	

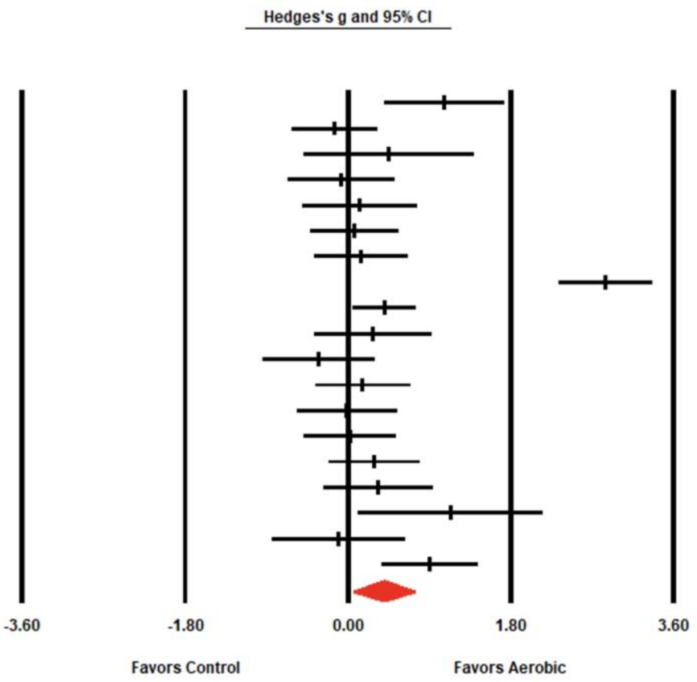

Supplementary Figure 2. Forest Plot of Individual Studies and Pooled Effects of Aerobic Exercise Interventions on Immediate Recall

Positive values of Hedges' *g* reflect improvements in immediate recall scores among those in the aerobic exercise group relative to the control group. A random-effects model was used. Results revealed a significant positive effect of aerobic exercise on immediate recall scores.

Study Name	Hedges's g	Standard error	Variance	Lower limit	Upper limit	Z-Value	p-Value	Relative weight
Antunes, 2015a	1.370	0.349	0.122	0.686	2.054	3.926	0.000	3.59
Antunes, 2015b	0.991	0.313	0.098	0.376	1.605	3.160	0.002	3.85
Ferreira, 2015	-0.334	0.298	0.089	-0.918	0.251	-1.120	0.263	3.97
Gaitán, 2019	0.492	0.409	0.167	-0.309	1.293	1.203	0.229	3.17
Hassmén, 1997	1.040	0.360	0.129	0.335	1.745	2.893	0.004	3.51
Ijuin, 2013	0.039	0.245	0.060	-0.442	0.520	0.159	0.874	4.37
Kramer, 2001	1.970	0.223	0.050	1.534	2.406	8.850	0.000	4.54
Lam, 2012	0.171	0.102	0.010	-0.030	0.371	1.671	0.095	5.30
Lam, 2015	-0.177	0.120	0.014	-0.412	0.059	-1.470	0.141	5.21
Lazarou, 2017	0.407	0.177	0.031	0.060	0.753	2.298	0.022	4.86
Legault, 2011	0.106	0.326	0.107	-0.534	0.745	0.323	0.746	3.76
Maass, 2015	-0.074	0.310	0.096	-0.682	0.535	-0.238	0.812	3.88
Maki, 2012	0.054	0.163	0.026	-0.264	0.373	0.334	0.738	4.96
Matura, 2017	-0.235	0.273	0.074	-0.770	0.300	-0.862	0.389	4.16
Morris, 2017	0.075	0.281	0.079	-0.477	0.626	0.265	0.791	4.10
Mortimer, 2012	0.193	0.255	0.065	-0.307	0.694	0.757	0.449	4.29
Nagamatsu, 2012	0.137	0.260	0.067	-0.372	0.645	0.527	0.598	4.26
Oken, 2006	0.000	0.208	0.043	-0.408	0.408	0.000	1.000	4.65
Scherder, 2005	-0.055	0.357	0.127	-0.753	0.644	-0.153	0.878	3.54
Tarumi, 2019	-0.706	0.245	0.060	-1.187	-0.226	-2.880	0.004	4.37
Thomas, 2020	0.652	0.365	0.133	-0.064	1.368	1.786	0.074	3.47
van Uffelen, 2008	-0.077	0.161	0.026	-0.393	0.240	-0.474	0.635	4.96
Varela, 2018	0.072	0.316	0.100	-0.548	0.693	0.229	0.819	3.83
Yogev-Seligmann 2021	-0.139	0.374	0.140	-0.872	0.595	-0.371	0.710	3.41
	0.232	0.111	0.012	0.014	0.451	2.085	0.037	

Statistics for each study

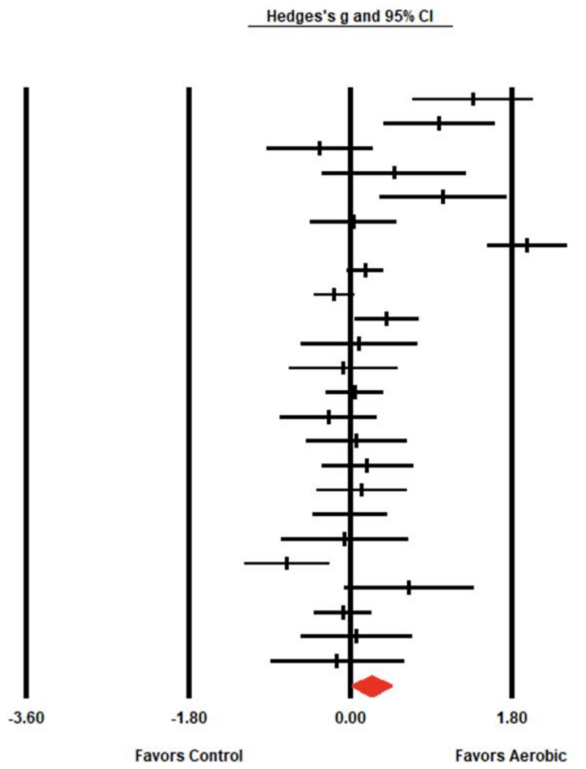

Supplementary Figure 3. Forest Plot of Individual Studies and Pooled Effects of Aerobic Exercise Interventions on Delayed Recall

Positive values of Hedges' *g* reflect improvements in delayed recall scores among those in the aerobic exercise group relative to the control group. A random-effects model was used. Results revealed a significant positive effect of aerobic exercise on delayed recall scores.

Study Name	Hedges's g	Standard error	Variance	Lower limit	Upper limit	Z-Value	p-Value	Relative weight
			Statistics for each study					
Jonasson, 2017	0.418	0.262	0.069	-0.095	0.932	1.597	0.110	14.40
Kramer, 2001	4.003	0.311	0.097	3.394	4.613	12.879	0.000	14.20
Maass, 2015	-0.398	0.314	0.098	-1.013	0.216	-1.270	0.204	14.19
Matura, 2017	-0.110	0.272	0.074	-0.643	0.423	-0.404	0.687	14.36
Mortimer, 2012	0.129	0.255	0.065	-0.371	0.629	0.505	0.613	14.43
Scherder, 2005	-0.095	0.355	0.126	-0.792	0.602	-0.268	0.789	14.00
ten Brinke, 2015	0.172	0.260	0.067	-0.337	0.681	0.663	0.507	14.41
	0.587	0.534	0.285	-0.459	1.633	1.099	0.272	

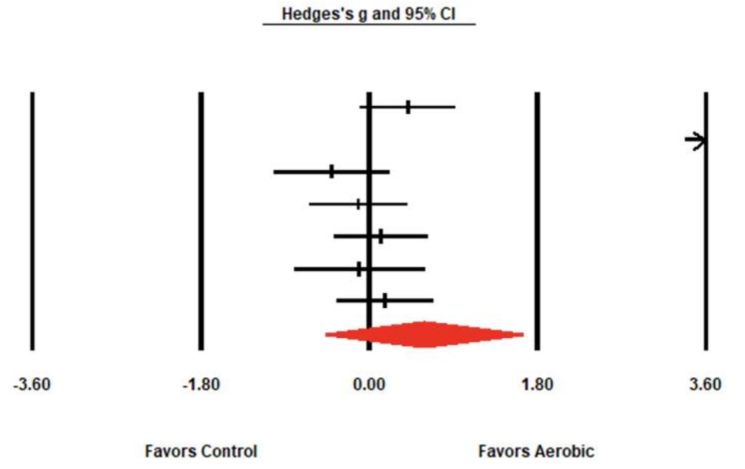

Supplementary Figure 4. Forest Plot of Individual Studies and Pooled Effects of Aerobic Exercise Interventions on Recognition

Positive values of Hedges' *g* reflect improvements in recognition scores among those in the aerobic exercise group relative to the control group. A random-effects model was used. Results did not reveal a significant effect of aerobic exercise on recognition scores.

Study Name	Hedges's g	Standard error	Variance	Lower limit	Upper limit	Z-Value	p-Value	Relative weight
Antunes, 2015b	1.562	0.333	0.111	0.910	2.213	4.696	0.000	3.25
Blumenthal, 1989	-0.006	0.240	0.057	-0.475	0.464	-0.024	0.981	3.84
Blumenthal, 2020	-0.339	0.225	0.050	-0.779	0.102	-1.508	0.132	3.93
Gaitán, 2019	0.434	0.407	0.166	-0.364	1.233	1.066	0.286	2.81
Hassmén, 1997	0.174	0.330	0.109	-0.472	0.820	0.528	0.598	3.27
Ijuin, 2013	0.065	0.245	0.060	-0.416	0.546	0.266	0.790	3.80
Jonasson, 2017	0.323	0.261	0.068	-0.188	0.835	1.238	0.216	3.70
Kramer, 2001	2.997	0.272	0.074	2.463	3.531	11.002	0.000	3.63
Lam, 2012	0.171	0.102	0.010	-0.030	0.371	1.671	0.095	4.56
Lam, 2015	-0.177	0.120	0.014	-0.412	0.059	-1.470	0.141	4.48
Lazarou, 2017	0.256	0.176	0.031	-0.089	0.601	1.456	0.145	4.21
Legault, 2011	0.072	0.326	0.107	-0.568	0.712	0.220	0.826	3.29
Maass, 2015	-0.264	0.312	0.097	-0.876	0.347	-0.847	0.397	3.38
Maki, 2012	0.054	0.163	0.026	-0.264	0.373	0.334	0.738	4.28
Matura, 2017	-0.060	0.272	0.074	-0.594	0.474	-0.219	0.826	3.63
Morris, 2017	0.150	0.282	0.079	-0.402	0.702	0.533	0.594	3.57
Mortimer, 2012	0.116	0.255	0.065	-0.384	0.616	0.453	0.651	3.74
Nagamatsu, 2012	0.203	0.260	0.068	-0.307	0.712	0.780	0.435	3.71
Oken, 2006	0.000	0.208	0.043	-0.408	0.408	0.000	1.000	4.03
Perri, 1985	0.342	0.306	0.094	-0.259	0.943	1.116	0.265	3.42
Ruscheweyh, 2011	0.465	0.311	0.097	-0.144	1.074	1.496	0.135	3.39
Scherder, 2005	-0.064	0.356	0.127	-0.761	0.633	-0.180	0.857	3.11
Stuckenschneider 2020	0.131	0.183	0.034	-0.228	0.490	0.718	0.473	4.17
Tarumi, 2019	-0.340	0.242	0.058	-0.814	0.133	-1.409	0.159	3.82
ten Brinke, 2015	0.172	0.260	0.067	-0.337	0.681	0.663	0.507	3.71
van Uffelen, 2008	-0.157	0.162	0.026	-0.474	0.160	-0.968	0.333	4.29
Yogev-Seligmann 2021	-0.155	0.374	0.140	-0.889	0.578	-0.415	0.678	3.00
	0.213	0.107	0.011	0.004	0.422	1.994	0.046	

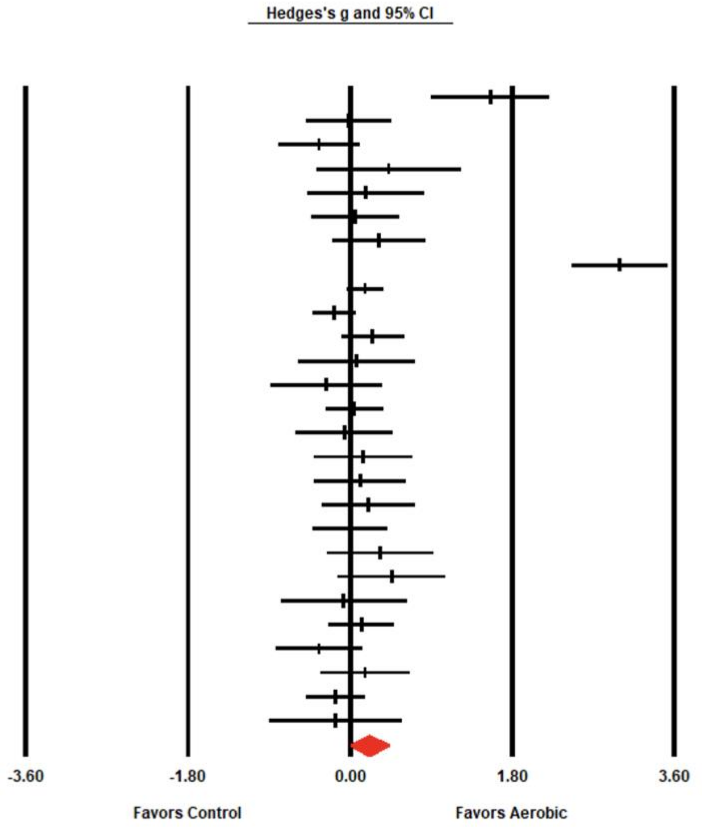

Supplementary Figure 5. Forest Plot of Individual Studies and Pooled Effects of Aerobic Exercise Interventions on Word-List Task Performance

Positive values of Hedges' *g* reflect improvements in word-list scores among those in the aerobic exercise group relative to the control group. A random-effects model was used. Results revealed a significant positive effect of aerobic exercise on word-list task performance.

Study Name	Hedges's g	Standard error	Variance	Lower limit	Upper limit	Z-Value	p-Value	Relative weight
Antunes, 2015a	1.221	0.342	0.117	0.550	1.892	3.566	0.000	7.69
Blumenthal, 1989	-0.144	0.239	0.057	-0.612	0.325	-0.601	0.548	10.35
Fabre, 2002	0.456	0.480	0.230	-0.484	1.396	0.952	0.341	5.18
Ferreira, 2015	-0.201	0.297	0.088	-0.784	0.381	-0.677	0.499	8.77
Ijuin, 2013	0.047	0.245	0.060	-0.434	0.528	0.191	0.849	10.17
Lazarou, 2017	0.407	0.177	0.031	0.060	0.754	2.302	0.021	12.17
Legault, 2011	0.141	0.327	0.107	-0.499	0.782	0.432	0.666	8.05
Morris, 2017	0.033	0.281	0.079	-0.518	0.585	0.119	0.906	9.19
Poinsatte, 2019	1.142	0.517	0.268	0.128	2.156	2.206	0.027	4.67
Thomas, 2020	0.652	0.365	0.133	-0.064	1.368	1.786	0.074	7.19
Yogev-Seligmann 2021	-0.081	0.374	0.140	-0.814	0.651	-0.218	0.827	7.01
Zhu, 2018	0.903	0.268	0.072	0.378	1.428	3.370	0.001	9.55
	0.334	0.134	0.018	0.072	0.597	2.497	0.013	

(Statistics for each study)

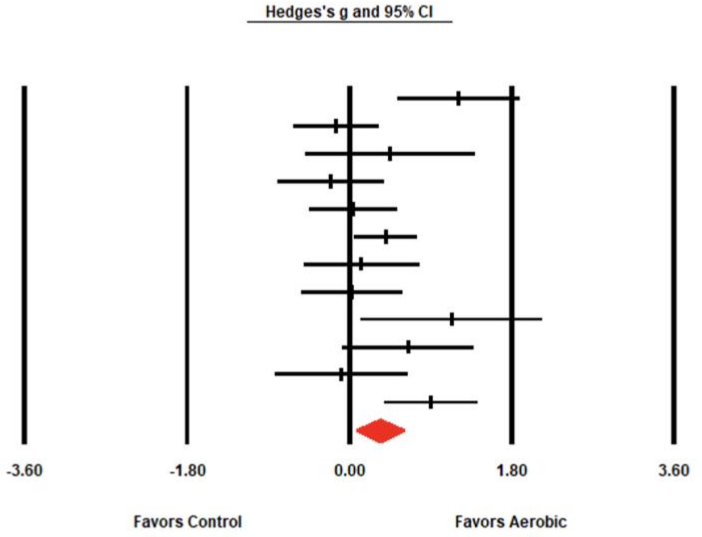

Supplementary Figure 6. Forest Plot of Individual Studies and Pooled Effects of Aerobic Exercise Interventions on Story-Recall Task Performance

Positive values of Hedges' *g* reflect improvements in story-recall scores among those in the aerobic exercise group relative to the control group. A random-effects model was used. Results revealed a significant positive effect of aerobic exercise on story-recall task performance.
