# Supplementary material for: Aerobic exercise improves episodic memory in late adulthood: a systematic review and meta-analysis
Source: Commun Med (Lond). 2022 Feb 17;2:15. doi: 10.1038/s43856-022-00079-7 (PMC9053291; doi:10.1038/s43856-022-00079-7)
Supplement: Supplementary file 7 — Reporting Summary [file 43856_2022_79_MOESM7_ESM.pdf]

nature research

Corresponding author(s): Sarah Aghjayan

Last updated by author(s): 01/14/2021

Reporting Summary

Nature Research wishes to improve the reproducibility of the work that we publish. This form provides structure for consistency and transparency in reporting. For further information on Nature Research policies, see our Editorial Policies and the Editorial Policy Checklist.

Statistics

For all statistical analyses, confirm that the following items are present in the figure legend, table legend, main text, or Methods section.

n/a	Confirmed	
☐	☒	The exact sample size (*n*) for each experimental group/condition, given as a discrete number and unit of measurement
☒	☐	A statement on whether measurements were taken from distinct samples or whether the same sample was measured repeatedly
☐	☒	The statistical test(s) used AND whether they are one- or two-sided<br>*Only common tests should be described solely by name; describe more complex techniques in the Methods section.*
☐	☒	A description of all covariates tested
☐	☒	A description of any assumptions or corrections, such as tests of normality and adjustment for multiple comparisons
☐	☒	A full description of the statistical parameters including central tendency (e.g. means) or other basic estimates (e.g. regression coefficient) AND variation (e.g. standard deviation) or associated estimates of uncertainty (e.g. confidence intervals)
☐	☒	For null hypothesis testing, the test statistic (e.g. *F*, *t*, *r*) with confidence intervals, effect sizes, degrees of freedom and *P* value noted<br>*Give P values as exact values whenever suitable.*
☒	☐	For Bayesian analysis, information on the choice of priors and Markov chain Monte Carlo settings
☒	☐	For hierarchical and complex designs, identification of the appropriate level for tests and full reporting of outcomes
☐	☒	Estimates of effect sizes (e.g. Cohen's *d*, Pearson's *r*), indicating how they were calculated

Our web collection on statistics for biologists contains articles on many of the points above.

Software and code

Policy information about availability of computer code

Data collection	Rayyan QCRI
Data analysis	Comprehensive Meta-Analysis software (CMA) Version 3

For manuscripts utilizing custom algorithms or software that are central to the research but not yet described in published literature, software must be made available to editors and reviewers. We strongly encourage code deposition in a community repository (e.g. GitHub). See the Nature Research guidelines for submitting code & software for further information.

Data

Policy information about availability of data

All manuscripts must include a data availability statement. This statement should provide the following information, where applicable:

- Accession codes, unique identifiers, or web links for publicly available datasets
- A list of figures that have associated raw data
- A description of any restrictions on data availability

Source data underlying all analyses, figures, and tables is available in Supplementary Data 2

Field-specific reporting

Please select the one below that is the best fit for your research. If you are not sure, read the appropriate sections before making your selection.

☐ Life sciences ☒ Behavioural & social sciences ☐ Ecological, evolutionary & environmental sciences

For a reference copy of the document with all sections, see nature.com/documents/nr-reporting-summary-flat.pdf

Behavioural & social sciences study design

All studies must disclose on these points even when the disclosure is negative.

Study description	Meta-analysis and systematic review examining the effects of aerobic exercise randomized controlled trials on episodic memory and whether characteristics of the sample and intervention moderate the effect.
Research sample	Adults with a mean age ≥55 years without dementia. All studies of participants with normal cognition (NC), subjective cognitive decline (SCD), and mild cognitive impairment (MCI) were included, regardless of the diagnostic criteria used. The average age ranged from 59-85 years, with a sample-size-weighted mean age of 70.82 years. Mean age was similar across exercise (70.64) and control groups (71.01). Females comprised 66.40% of the participants across all studies. Twenty studies included individuals with NC and 16 studies included individuals with impaired cognition, consisting of 1 SCD and 15 MCI studies.
Sampling strategy	A literature search was conducted using the following databases: PubMed, CINAHL, EMBASE, PsycINFO, and CENTRAL. The following combination of search terms was used: memory OR recall OR verbal learning test OR reminding test OR story recall OR list learning OR word list OR paired associations AND randomized control trial* OR clinical trial OR RCT AND exercise* OR physical activity OR physical training AND aerobic AND older adult* OR aging OR aged OR elderly OR geriatric OR cognitive impairment OR cognitive decline OR memory decline. . We identified additional titles by a manual search of relevant journals (International Journal of Sports Medicine, Frontiers in Aging Neuroscience, and British Journal of Sports Medicine) and by identifying references in the six aforementioned meta-analyses.
Data collection	The first author screened all returned titles to exclude duplicate studies. The first and second authors independently screened the remaining 699 titles and abstracts for eligibility based on the inclusion criteria described above. The authors agreed on the eligibility decision for 673 articles (96%); the first author resolved disagreements on the remaining 26. If articles appeared to be eligible but did not provide enough data to estimate an effect size or enough information about the intervention, the first author contacted the study's corresponding author (k=9). All data were extracted and coded by the first author. All extracted variables were reviewed by the second author to ensure data accuracy.
Timing	A literature search was conducted on April 1st, 2021.
Data exclusions	Based on the pre-established exclusionary criteria, studies were excluded for the following reasons:<br>Sample (n = 223)<br>Study design (n = 208)<br>Did not measure episodic memory (n = 79)<br>Did not include only aerobic exercise (n = 80)<br>Included strength training (n = 23)<br>Not a peer-reviewed journal article (n = 19)<br>No control group (n = 12)<br>Cognitive status (n=9)<br>Duplicate data (n = 6)<br>Strength training in aerobic group (n = 1)<br>Cognitive training in control group (n = 1)<br>Wrong study design (n = 1)
Non-participation	The included studies did not consistently report withdrawal/attrition and attendance/adherence, limiting our ability to systematically examine whether these key intervention characteristics impact the effect of aerobic exercise on episodic memory.
Randomization	Subjects were randomly allocated to groups.

Reporting for specific materials, systems and methods

We require information from authors about some types of materials, experimental systems and methods used in many studies. Here, indicate whether each material, system or method listed is relevant to your study. If you are not sure if a list item applies to your research, read the appropriate section before selecting a response.

Materials & experimental systems

n/a	Involved in the study
☒	☐ Antibodies
☒	☐ Eukaryotic cell lines
☒	☐ Palaeontology and archaeology
☒	☐ Animals and other organisms
☒	☐ Human research participants
☒	☐ Clinical data
☒	☐ Dual use research of concern

Methods

n/a	Involved in the study
☒	☐ ChIP-seq
☒	☐ Flow cytometry
☒	☐ MRI-based neuroimaging
